# Adaptive Fuzzy Sliding Mode Control for a Micro Gyroscope with Backstepping Controller

**DOI:** 10.3390/mi11110968

**Published:** 2020-10-29

**Authors:** Juntao Fei, Yunmei Fang, Zhuli Yuan

**Affiliations:** 1 College of IoT Engineering, Hohai University, Changzhou 213022, China; 2 Jiangsu Key Laboratory of Power Transmission and Distribution Equipment Technology, Changzhou 213022, China; yunmeif@163.com (Y.F.); bigboyscn@yahoo.com (Z.Y.)

**Keywords:** adaptive control, backstepping approach, tracking performance, micro gyroscope

## Abstract

This paper developed an adaptive backstepping fuzzy sliding control (ABFSC) approach for a micro gyroscope. Based on backstepping design, an adaptive fuzzy sliding mode control was proposed to adjust the fuzzy parameters with self-learning ability and reject the system nonlinearities. With the Lyapunov function analysis of error function and sliding surface function, a comprehensive controller is derived to ensure the stability of the proposed control system. The proposed fuzzy control scheme does not need to know the system model in advance and could approximate the system nonlinearities well. The adaptive fuzzy control method has self-learning ability to adjust the fuzzy parameters. Simulation studies were implemented to prove the validity of the proposed ABFSMC strategy, showing that it can adapt to the changes of external disturbance and model parameters and has a satisfactory performance in tracking and approximation.

## 1. Introduction

Micro gyroscopes are widely used in inertial navigation and guidance systems. However the error and disturbances existing in micro gyroscopes may decrease the accuracy and sensitivity of the system. With the advancement of microelectromechanical system (MEMS) technology, it is possible to produce chip-based sensors such as accelerometers, gyroscopes, and magnetometers, in combination with the miniaturization of electronic devices. Micro gyroscopes are small, lightweight, have low energy consumption, long service life, and are extremely low cost.

Many control strategies have been investigated to compensate the error and disturbance from micro gyroscopes [1,2,3,4,5,6,7]. Some intelligent controllers are widely used in dynamic systems because of their good capacity to approximate any unknown smooth functions [8,9,10,11,12]. Adaptive sliding mode controllers are combined with intelligent controllers for dynamic systems [13,14,15,16,17]. Thee backstepping method is widely used in dynamic systems with pure feedback or strict feedback forms. The backstepping adaptive fuzzy control method has received great interest in recent years [18]. Neural control and fuzzy control have the capacity to approximate unknown smooth functions and have been widely used in identification and control [19,20,21,22,23,24,25,26,27,28,29,30].

The universal approximation theorem indicates that the fuzzy system is a new universal approximator in addition to polynomial function approximators and neural network approximators. As the universal approximation theory of fuzzy system can approximate any nonlinear model and realize arbitrary nonlinear control law, it is widely used in control systems. Adaptive sliding mode control with a neural estimator and adaptive control with fuzzy compensator for a micro gyroscope was investigated in [31,32]. In [33], a fuzzy system was used to approximate the system nonlinearities and a finite time convergent sliding mode controller was designed. Backstepping design is a powerful tool for dynamic systems with pure or strict feedback forms. In recent years, the backstepping control techniques have received great attention due to their systematic and recursive design methodology for nonlinear feedback control. The key idea of the backstepping technique is to recursively select some appropriate functions of state variables as fictitious control inputs for lower dimension subsystems of the overall system. Lin et al. [34] and Lee et al. [35] introduced an adaptive fuzzy backstepping control method for uncertain nonlinear systems. Lin et al. [36] proposed an adaptive fuzzy sliding-mode control for linear ultrasonic servomotor systems.

However, the fuzzy control strategy is not combined with an adaptive backstepping controller in the application of micro gyroscope and a backstepping controller has not been incorporated into a fuzzy sliding mode control system for a micro gyroscope. As such, the adaptive backstepping fuzzy sliding mode control approach has not been proposed in the control of a micro gyroscope. Motivated by the above research, this paper presents an adaptive backstepping fuzzy sliding controller to adjust the fuzzy parameters with self-learning ability and reject the system nonlinearities. Comparing other existing methods, the main contributions of this article can be summarized as follows:

(1) Backstepping is a nonlinear control approach by means of the recursion process. A major advantage of a backstepping controller is its flexibility to avoid cancellations of useful nonlinearities and achieve regulation and tracking properties. The gyroscope equations are transformed into an analogically cascade system where the backstepping approach can be implanted.

(2) Backstepping design and adaptive fuzzy sliding mode control were applied to a micro gyroscope. The proposed adaptive fuzzy controller not only does not rely on the system model, but also makes the algorithm adjust the fuzzy parameters with self-learning ability.

(3) The proposed sliding mode control adds additional compensators to improve the stability, hence obtaining desired system characteristics. Thus, the entire closed-loop system meets the dynamic and static performance indicators and achieves accurate tracking performance.

## 2. Dynamics of Micro Gyroscope

**Figure 1:**
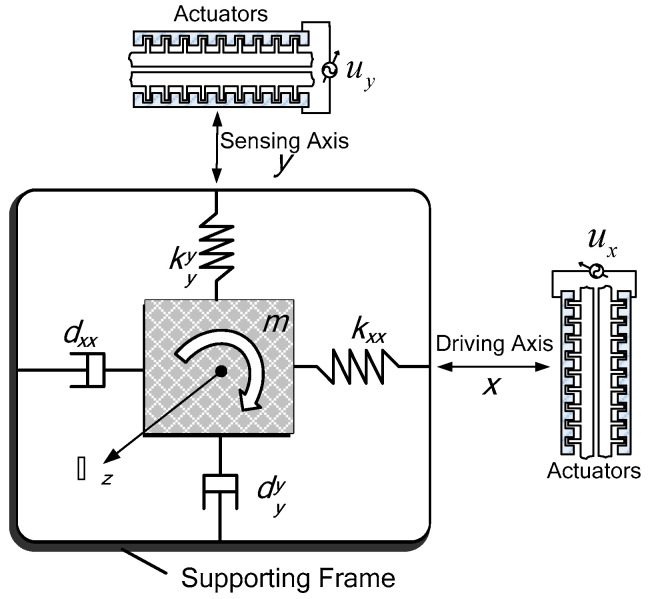
Schematic diagram of a micro gyroscope.

In this section, the dynamic model of a micro gyroscope is presented. The characteristics of micro gyroscopes are similar to traditional gyroscopes, mainly through the Coriolis force to achieve the miniaturization of equipment. The dynamics model of the micro gyroscope can be regarded as a damping-spring-mass system, as shown in Figure 1.

The driving electrode generates electrostatic force to drive the base mass block back and forth to maintain stable oscillatory momentum. The induction device is used to sense the movement of the base mass block in the vertical driving direction, and to extract the external angular velocity from the vibration information. Referring to Park [1], with some assumptions, the dynamic model of gyroscope can be expressed as

(1) $$\begin{array}{l} m\ddot{x}+d_{xx}\dot{x}+d_{xy}\dot{y}+k_{xx}x+k_{xy}y=u_{x}+2m\Omega_{z}\dot{y}\\ m\ddot{y}+d_{xy}\dot{x}+d_{yy}\dot{y}+k_{xy}x+k_{yy}y=u_{y}-2m\Omega_{z}\dot{x} \end{array}$$

The asymmetric spring and damping terms, $k_{xy}$ and $d_{xy}$, are generated from fabrication imperfections. $k_{xx}$, $k_{yy}$, $d_{xx}$, and $d_{yy}$ are the x and y axes spring and damping terms. $u_{x}$ and $u_{y}$ are the control forces.

Dividing both sides of Equation (1) by the mass m, reference length $q_{0}$, and the square of the resonance frequency $w_{0}^{2}$ yields

$$\frac{d_{xx}}{mw_{0}^{2}}\to D_{xx},\frac{d_{xy}}{mw_{0}^{2}}\to D_{xy},\frac{d_{yy}}{mw_{0}^{2}}\to D_{yy},\frac{k_{xx}}{mw_{0}^{2}}\to w_{x}^{2},\frac{k_{xy}}{mw_{0}^{2}}\to w_{xy},\frac{k_{yy}}{mw_{0}^{2}}\to w_{y}^{2},\frac{\Omega_{z}}{w_{0}^{2}}\to \Omega_{Z}$$

Define $x_{1}=\left[\begin{array}{cc} x & y \end{array}\right]$, Equation (1) can be rewritten as

(2) $$\{\begin{array}{c} {\dot{x}}_{1}=x_{2}\\ {\dot{x}}_{2}=-(D+2\Omega )x_{2}-K_{b}x_{1}+u \end{array}$$

where the dimensionless quantities are

$$D=\left[\begin{array}{cc} D_{xx} & D_{xy}\\ D_{xy} & D_{yy} \end{array}\right],K_{b}=\left[\begin{array}{cc} w_{x}^{2} & w_{xy}\\ w_{xy} & w_{y}^{2} \end{array}\right],u=\left[\begin{array}{c} u_{x}\\ u_{y} \end{array}\right],\Omega =\left[\begin{array}{cc} 0 & -\Omega_{Z}\\ \Omega_{Z} & 0 \end{array}\right]$$

Taking into account the system nonlinearities, Equation (2) can be expressed as:

(3) $$\begin{array}{cc} {\dot{x}}_{2} & =[-(D+2\Omega )+\Delta A_{1}]x_{2}+(-K_{b}+\Delta A_{2})x_{1}+(I+\Delta B)u+\eta \\ & =-(D+2\Omega )x_{2}-Kx_{1}+u+H(t) \end{array}$$

where $\Delta A_{1},\Delta A_{2},\Delta B$ are the model uncertainties; and η is external disturbances of micro gyroscope. H(t) includes lumped system nonlinearities, $H(t)=\Delta A_{1}x_{2}+\Delta A_{2}x_{1}+\Delta Bu+\eta$. We assume ‖H(t)‖ is bounded by a positive constant $H_{\max}$.

## 3. Adaptive Backstepping Control Design

**Figure 2:**
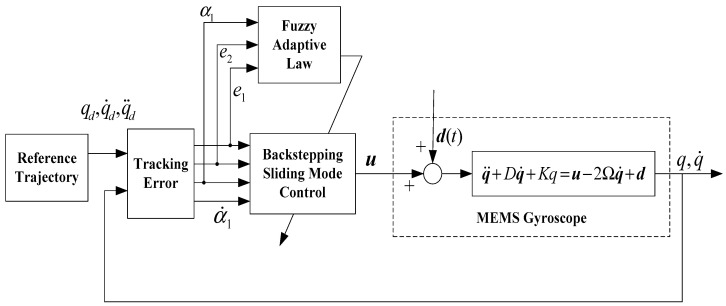
Block diagram of the proposed adaptive backstepping fuzzy sliding control (ABFSMC).

Motivated by the research results in [17,18,19,20,21,28], in this section, a backstepping controller was designed to meet the objective of tracking and stabilization by a recursive design procedure. The schematic block diagram of the proposed approach of a micro gyroscope is described in Figure 2. The target of ABFSMC is to achieve real-time compensation for fabrication imperfections.

The reference model is defined as r. The tracking error is defined as

(4) $$\{\begin{array}{c} e_{1}=x_{1}-r\\ e_{2}=x_{2}-\alpha_{1} \end{array}$$

where r is the trajectory of the reference model virtual control volume and $\alpha_{1}$ is the virtual control volume, defined as

(5) $$\alpha_{1}=-c_{1}e_{1}+\dot{r}$$

In Equation (5), $c_{1}$ is a positive constant.

We selected the first Lyapunov function as:

(6) $$V_{1}=\frac{1}{2}e_{1}^{T}e_{1}$$

The time derivative of the $V_{1}$ is

(7) $${\dot{V}}_{1}=e_{1}^{T}{\dot{e}}_{1}=e_{1}^{T}(x_{2}-\dot{r})=e_{1}^{T}(e_{2}-c_{1}e_{1})=-c_{1}e_{1}^{T}e_{1}+e_{1}^{T}e_{2}$$

when $e_{2}=0$, it is easy to see that ${\dot{V}}_{1}=-c_{1}e_{1}^{T}e_{1}\leq 0$.

Define a function of sliding surface as

(8) $$s=ce_{1}+e_{2}$$

where c is a positive constant.

Define the second Lyapunov function as

(9) $$V_{2}=V_{1}+\frac{1}{2}s^{T}s$$

From Equation (9), we can get

(10) $$e_{1}=\frac{1}{c}(s-e_{2})$$

Then, substituting Equation (10) into the time derivative of $V_{2}$ becomes

(11) $$\begin{array}{cc} {\dot{V}}_{2} & =-c_{1}e_{1}^{T}e_{1}+e_{1}^{T}e_{2}+s^{T}(c{\dot{e}}_{1}+{\dot{e}}_{2})\\ & =-c_{1}e_{1}^{T}e_{1}+e_{1}^{T}e_{2}+s^{T}[c(x_{2}-\dot{r})+{\dot{x}}_{2}-{\dot{\alpha }}_{1}]\\ & =-c_{1}e_{1}^{T}e_{1}-\frac{1}{c}e_{2}^{T}e_{2}+s^{T}[(c+\frac{1}{c})e_{2}+c(\alpha_{1}-\dot{r})+u+H(t)-{\dot{\alpha }}_{1}+f(x_{1},x_{2})] \end{array}$$

where $f(x_{1},x_{2})=-(D+2\Omega )x_{2}-Kx_{1}$.

In the controller design, we used exponential reaching law as

(12) $$\dot{s}=-\rho s-H_{\max}\mathrm{sgn}(s)$$

where ρ>0.

By Equations (11) and (12), we designed a comprehensive controller as

(13) $$u=-[(\frac{1}{c}+c)e_{2}+c(\alpha_{1}-\dot{r})-{\dot{\alpha }}_{1}]-f(x_{1},x_{2})-\rho s-H_{\max}\mathrm{sgn}(s)$$

Substituting Equation (13) into Equation (11) yields

(14) $$\begin{array}{cc} {\dot{V}}_{2} & =-c_{1}e_{1}^{T}e_{1}-ce_{2}^{T}e_{2}+s^{T}(H(t)-H_{\max}\mathrm{sgn}(s))-\rho s^{T}s\\ & \leq -c_{1}e_{1}^{T}e_{1}-ce_{2}^{T}e_{2}+\left\|s\right\|(\left\|H(t)\right\|-H_{\max})-\rho s^{T}s\\ & \leq 0 \end{array}$$

However, since the system model $f(x_{1},x_{2})$ is unknown in practical situations, the controller (13) cannot be implemented. Then, a fuzzy system is used to approximate the unknown model of the micro gyroscope.

The singleton fuzzifier mapping was used, where $g_{i}$ and $\hat{f}$ have the same member functions as Gaussian membership functions

(15) $$\mu_{A_{i}^{l}}\left(g_{i}\right)=\exp\left(-\frac{{\left(g_{i}-c_{i}\right)}^{2}}{2\sigma_{i}^{2}}\right)$$

where $c_{i}$ and $\sigma_{i}$ are the center and width of the ith fuzzy set $A_{i}^{l}$, respectively.

The output of the fuzzy system is written by a center-average defuzzifier, product inference, and singleton fuzzifier as:

(16) $${\hat{f}}^{T}(g)=\frac{\sum_{l=1}^{M}h_{l}\left(\prod_{i=1}^{n}\mu_{A_{i}^{l}}\left(g_{i}\right)\right)}{\sum_{l=1}^{M}\left(\prod_{i=1}^{n}\mu_{A_{i}^{l}}\left(g_{i}\right)\right)}=\theta^{T}\xi (g)$$

where $\mu_{A_{i}^{l}}\left(g_{i}\right)$ is the membership function value of the fuzzy variable $g_{i}$; $d_{l}$ is the point at which the membership function of $B^{l}$ achieves its maximum value; $\theta^{T}=\left(\begin{array}{cccc} \theta_{1}, & \theta_{2}, & \cdots , & \theta_{M} \end{array}\right)$ is the adaptive parameter, and $\xi \left(g\right)={\left(\begin{array}{cccc} \xi_{1}(g), & \xi_{2}(g), & \cdots , & \xi_{M}(g) \end{array}\right)}^{T}$ are the fuzzy basis functions.

We defined the optimal approximation constant $\theta^{*}$. We made an assumption that for a given small arbitrarily positive constant ε, the following inequality expression (17) holds.

(17) $$\left\|f-\xi^{T}(g)\theta^{*}\right\|\leq \varepsilon$$

Since the system model $f(x_{1},x_{2})$ is unknown, a fuzzy controller can be proposed as

(18) $$u=-[(\frac{1}{c}+c)e_{2}+c(\alpha_{1}-\dot{r})-{\dot{\alpha }}_{1}]-\hat{f}-\rho s-H_{\max}\mathrm{sgn}(s)$$

where a fuzzy system $\hat{f}=\xi^{T}(x)\theta$ is used to approximate the $f(x_{1},x_{2})$ in (13).

## 4. Adaptive Estimator

In this section, the stability analysis of the proposed control system is discussed. First, we chose a Lyapunov function candidate as

(19) $$V=\frac{1}{2}e_{1}^{T}e_{1}+\frac{1}{2}s^{T}s+\frac{1}{2\tau }{\tilde{\theta }}^{T}\tilde{\theta }$$

where τ>0, $\tilde{\theta }=\theta^{*}-\theta$.

Then, the time derivative of V becomes

(20) $$V=e_{1}^{T}{\dot{e}}_{1}+s^{T}\dot{s}+\frac{1}{\tau }{\dot{\tilde{\theta }}}^{T}\tilde{\theta }$$

From the derivation in (11),

(21) $$\begin{array}{cc} {\dot{V}}_{2} & =-c_{1}e_{1}^{T}e_{1}+e_{1}^{T}e_{2}+s^{T}(c{\dot{e}}_{1}+{\dot{e}}_{2})-\frac{1}{\tau }{\tilde{\theta }}^{T}\dot{\theta }\\ & =-c_{1}e_{1}^{T}e_{1}+e_{1}^{T}e_{2}+s^{T}[c(x_{2}-\dot{r})+{\dot{x}}_{2}-{\dot{\alpha }}_{1}]-\frac{1}{\tau }{\tilde{\theta }}^{T}\dot{\theta }\\ & =-c_{1}e_{1}^{T}e_{1}-\frac{1}{c}e_{2}^{T}e_{2}+s^{T}[(c+\frac{1}{c})e_{2}+c(\alpha_{1}-\dot{r})+u+H(t)-{\dot{\alpha }}_{1}+f(x_{1},x_{2})]-\frac{1}{\tau }{\tilde{\theta }}^{T}\dot{\theta } \end{array}$$

Substituting (18) into (21) yields

(22) $$\begin{array}{cc} \dot{V} & =-c_{1}e_{1}^{T}e_{1}-ce_{2}^{T}e_{2}+s^{T}(H(t)-H_{\max}\mathrm{sgn}(s))+s^{T}(f(x,y)-\hat{f})-\rho s^{T}s-\frac{1}{\tau }{\tilde{\theta }}^{T}\dot{\theta }\\ & =-c_{1}e_{1}^{T}e_{1}-ce_{2}^{T}e_{2}+s^{T}(H(t)-H_{\max}\mathrm{sgn}(s))+s^{T}(f(x,y)-\xi^{T}(x)\theta )-\rho s^{T}s-\frac{1}{\tau }{\tilde{\theta }}^{T}\dot{\theta }\\ & =-c_{1}e_{1}^{T}e_{1}-ce_{2}^{T}e_{2}+s^{T}(H(t)-H_{\max}\mathrm{sgn}(s))+s^{T}(f(x,y)-\xi^{T}(x)\theta^{*})+s^{T}(\xi^{T}(x)\theta^{*}-\xi^{T}(x)\theta )-\rho s^{T}s-\frac{1}{\tau }{\tilde{\theta }}^{T}\dot{\theta } \end{array}$$

Since ‖H(t)‖ is bounded by a positive constant $H_{\max}$, and making using of $ab\leq \frac{1}{2}a^{2}+\frac{1}{2}b^{2}$ and (17), (22) becomes

(23) $$\begin{array}{cc} \dot{V} & \leq -c_{1}e_{1}^{T}e_{1}-ce_{2}^{T}e_{2}+\left\|s\right\|(H_{\max}-H(t))+s^{T}(f(x,y)-\xi^{T}(x)\theta^{*})+s^{T}(\xi^{T}(x)\theta^{*}-\xi^{T}(x)\theta )-\rho s^{T}s-\frac{1}{\tau }{\tilde{\theta }}^{T}\dot{\theta }\\ & \leq -c_{1}e_{1}^{T}e_{1}-ce_{2}^{T}e_{2}+s^{T}(f(x,y)-\xi^{T}(x)\theta^{*})+s^{T}(\xi^{T}(x)\theta^{*}-\xi^{T}(x)\theta )-\rho s^{T}s-\frac{1}{\tau }{\tilde{\theta }}^{T}\dot{\theta }\\ & \leq c_{1}e_{1}^{T}e_{1}-ce_{2}^{T}e_{2}+\frac{1}{2}{\left\|s\right\|}^{2}+\frac{1}{2}\varepsilon^{2}-\rho s^{T}s-{\tilde{\theta }}^{T}\left[{\left(s^{T}\xi^{T}(x)\right)}^{T}-\frac{1}{\tau }\dot{\theta }\right] \end{array}$$

To make ${\dot{V}}_{2}\leq 0$, we chose a parameter updating law

(24) $$\dot{\theta }=\tau {\left(s^{T}\xi^{T}(x)\right)}^{T}+2\gamma \theta$$

Substituting Equation (24) into Equation (23) yields

(25) $$\begin{array}{cc} \dot{V} & \leq -c_{1}e_{1}^{T}e_{1}-ce_{2}^{T}e_{2}+\frac{1}{2}{\left\|s\right\|}^{2}+\frac{1}{2}\varepsilon^{2}-\rho s^{T}s-\frac{2\gamma }{\tau }{\tilde{\theta }}^{T}\theta \\ & =c_{1}e_{1}^{T}e_{1}-ce_{2}^{T}e_{2}+\frac{1}{2}{\left\|s\right\|}^{2}+\frac{1}{2}\varepsilon^{2}-\rho s^{T}s-\frac{\gamma }{\tau }(2\theta^{*T}\theta -2\theta^{T}\theta ) \end{array}$$

According to the Inequality ${\left(\theta -\theta^{*}\right)}^{T}(\theta -\theta^{*})\geq 0$, we can get $2\theta^{*T}\theta -2\theta^{T}\theta \leq -\theta^{T}\theta +\theta^{*T}\theta^{*}$. Substituting this condition into Equation (25) yields

(26) $$\begin{array}{cc} \dot{V} & \leq c_{1}e_{1}^{T}e_{1}-ce_{2}^{T}e_{2}+\frac{1}{2}{\left\|s\right\|}^{2}+\frac{1}{2}\varepsilon^{2}-\rho s^{T}s-\frac{\gamma }{\tau }(-\theta^{T}\theta +\theta^{*T}\theta^{*})\\ & =c_{1}e_{1}^{T}e_{1}-ce_{2}^{T}e_{2}+\frac{1}{2}{\left\|s\right\|}^{2}+\frac{1}{2}\varepsilon^{2}-\rho s^{T}s+\frac{\gamma }{\tau }(\theta^{T}\theta +\theta^{*T}\theta^{*})-\frac{2\gamma }{\tau }\theta^{*T}\theta^{*} \end{array}$$

According to the inequality ${\left(\theta +\theta^{*}\right)}^{T}(\theta +\theta^{*})\geq 0$, that is $-\theta^{*T}\theta -\theta^{T}\theta^{*}\leq \theta^{*T}\theta^{*}+\theta^{T}\theta$, we can get

(27) $$\begin{array}{cc} -\frac{1}{2}{\tilde{\theta }}^{T}\tilde{\theta } & =-\frac{1}{2}{\left(\theta^{*}-\theta \right)}^{T}(\theta^{*}-\theta )\\ & =-\frac{1}{2}(\theta^{*T}\theta^{*}+\theta^{T}\theta -\theta^{*T}\theta -\theta^{T}\theta^{*})\\ & \leq \theta^{*T}\theta^{*}+\theta^{T}\theta \end{array}$$

Substituting Equation (27) into Equation (26) yields

(28) $$\begin{array}{cc} \dot{V} & \leq c_{1}e_{1}^{T}e_{1}-ce_{2}^{T}e_{2}+\frac{1}{2}{\left\|s\right\|}^{2}+\frac{1}{2}\varepsilon^{2}-\rho s^{T}s-\frac{\gamma }{\tau }(\frac{1}{2}{\tilde{\theta }}^{T}\tilde{\theta })-\frac{2\gamma }{\tau }\theta^{*T}\theta^{*}\\ & =-\frac{2}{2}c_{1}e_{1}^{T}e_{1}-\frac{2}{2}(\rho -\frac{1}{2})s^{T}s-\frac{\gamma }{2\tau }{\tilde{\theta }}^{T}\tilde{\theta }-\frac{1}{c}e_{2}^{T}e_{2}+\frac{1}{2}\varepsilon^{2}-\frac{2\gamma }{\tau }\theta^{*T}\theta^{*} \end{array}$$

where ρ>1/2.

Define $c_{0}=\min\left\{2c_{1},2(\rho -1/2),\gamma \right\}$, Equation (28) becomes

(29) $$\begin{array}{cc} \dot{V} & \leq -\frac{c_{0}}{2}(e_{1}^{T}e_{1}+s^{T}s+\frac{1}{\tau }{\tilde{\theta }}^{T}\tilde{\theta })-\frac{1}{c}e_{2}^{T}e_{2}+\frac{1}{2}\varepsilon^{2}-\frac{2\gamma }{\tau }\theta^{*T}\theta^{*}\\ & =-c_{0}V-\frac{1}{c}e_{2}^{T}e_{2}+\frac{1}{2}\varepsilon^{2}-\frac{2\gamma }{\tau }\theta^{*T}\theta^{*}\\ & =-c_{0}V+c_{V\max} \end{array}$$

where $c_{V\max}=-\frac{1}{c}e_{2}^{T}e_{2}+\frac{1}{2}\varepsilon^{2}-\frac{2\gamma }{\tau }\theta^{*T}\theta^{*}$.

Solving Equation (29) yields

(30) $$\begin{array}{cc} V(t) & \leq V(0)\exp(-c_{0}t)+\frac{c_{V\max}}{c_{0}}\left(1-\exp(-c_{0}t)\right)\\ & \leq V(0)+\frac{c_{V\max}}{c_{0}}\left(\forall t\geq 0\right) \end{array}$$

where V(0) is the initial value of V. If we define a closely collection as $\Omega_{0}=\left\{X|V(X)\leq V(0)+\frac{c_{V\max}}{c_{0}}\right\}$, we can get $\left\{e_{1},s,\tilde{\theta }\right\}\in \Omega_{0}$. $\dot{V}$ is a negative semi-definite that ensures that V, $e_{1}$, s, $\tilde{\theta }$ are all bounded. Then, the stability of the designed closed-loop control system can be guaranteed.

**Remark** **1.** *In order to overcome this problem, a proper adaptation law can be proposed to estimator the upper bound* $H_{\max}$ *to realize the adaptive upper bound control, weakening the chattering and ensuring the stability of the control system.*

**Remark** **2.** *Since there is a switching functions sign in the proposed controller (13), we can use saturation function or hyperbolic function to replace sign function to decrease chattering.*

## 5. Simulation Study

In this section, the proposed ABFSMC scheme was evaluated on the lumped model of a micro gyroscope sensor. The simulation experiment of the proposed scheme was carried out on a MATLAB/SIMULINK software platform. Meanwhile, the superiority of the proposed can be further confirmed by comparing it with the adaptive backstepping controller [7].

The parameters of the micro gyroscope were selected as in Table 1.

The angular velocity of micro gyroscope was assumed to be $\Omega_{z}=100\;\mathrm{rad}/s$. The dimensionless procedure was implemented. The reference length and frequency were chosen as $q_{0}=1\;\mu m$ and $\omega_{0}=1000\;\mathrm{Hz}$. The unknown angular velocity was assumed to be $\Omega_{z}=100\;\mathrm{rad}/s$, and the dimensionless parameters can be calculated. The reference trajectory $r_{1}=\sin(4.17t)$, $r_{2}=1.2\sin(5.11t)$, was close to their natural frequencies. Random variable signal with zero mean and unity variance was regarded as external disturbance H(t). As for model uncertainties, we assumed there existed ±30% parameter variations for the spring and damping coefficients, and ±30% magnitude changes held in the coupling terms with respect to their nominal values.

Initial conditions were $q(0)={\left[\begin{array}{cc} 1 & 1 \end{array}\right]}^{T}$, other parameters were selected as c=15, $c_{1}=10$, $H_{\max}=1000$, τ=2, γ=1.5, ρ=20. The membership functions were selected as

$$\mu_{F_{i}}^{1}=\exp[-0.5{\left((x_{i}+A_{i}/2)/(A_{i}/4)\right)}^{2}],\;\mu_{F_{i}}^{2}=\exp[-0.5{\left(x_{i}/(A_{i}/4)\right)}^{2}],\;\mu_{F_{i}}^{3}=\exp[-0.5{\left((x_{i}-A_{i}/2)/(A_{i}/4)\right)}^{2}],\;(i=1,2,3,4).$$

where $A_{i}$ is the amplitude of the reference trajectory as $\left[\begin{array}{cccc} 1 & 1.2 & 4.17 & 6.132 \end{array}\right]$.

**Figure 3:**
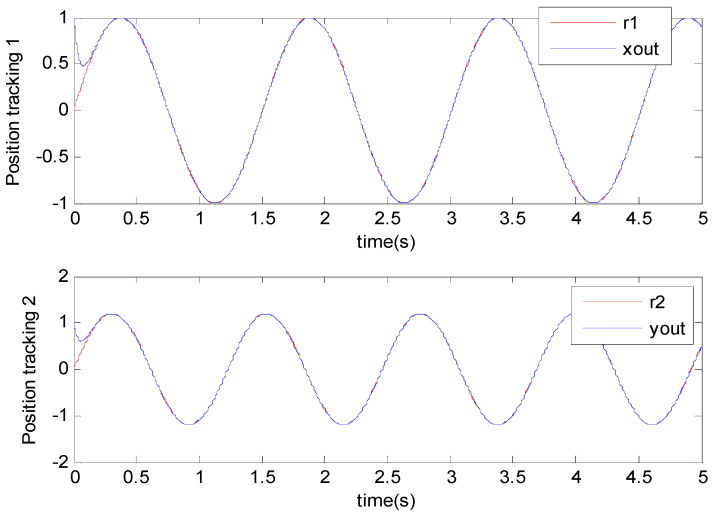
Trajectory tracking using the ABFSMC approach.

**Figure 4:**
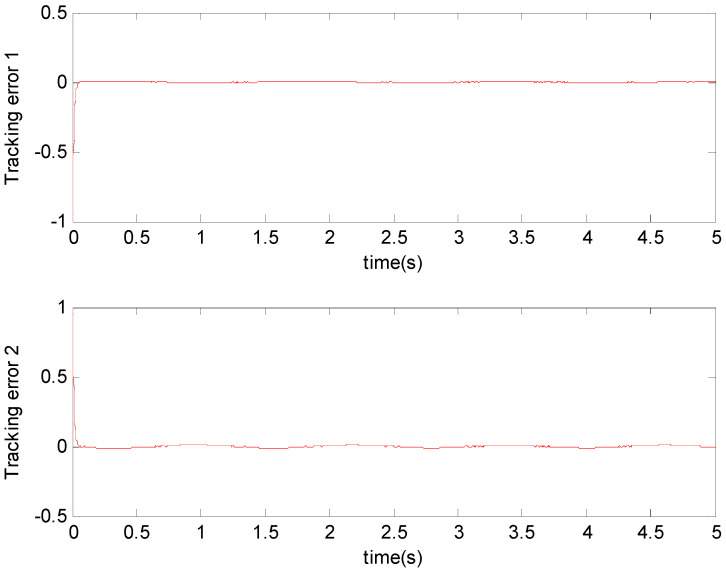
Tracking error using the ABFSMC approach.

**Figure 5:**
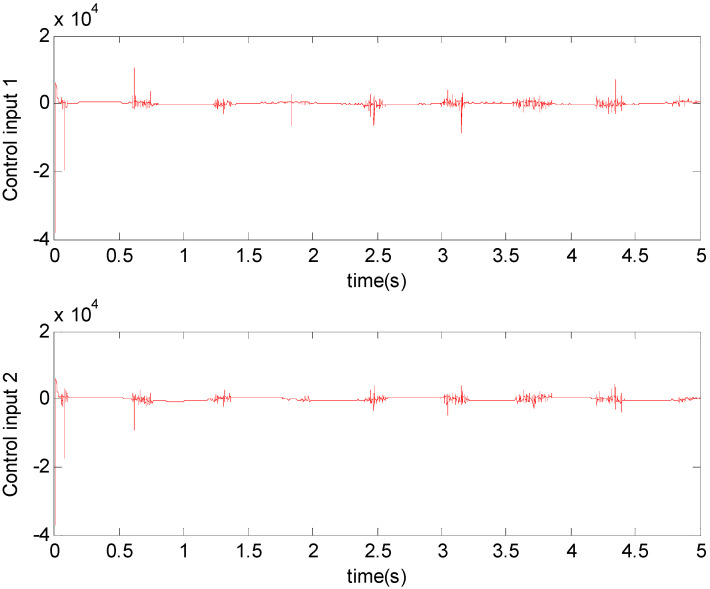
Control input using the ABFSMC approach.

**Figure 6:**
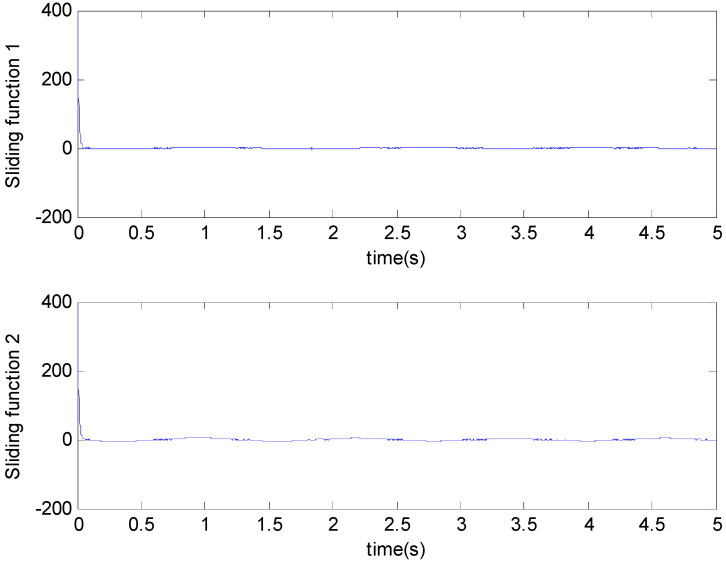
Sliding mode surface function.

**Figure 7:**
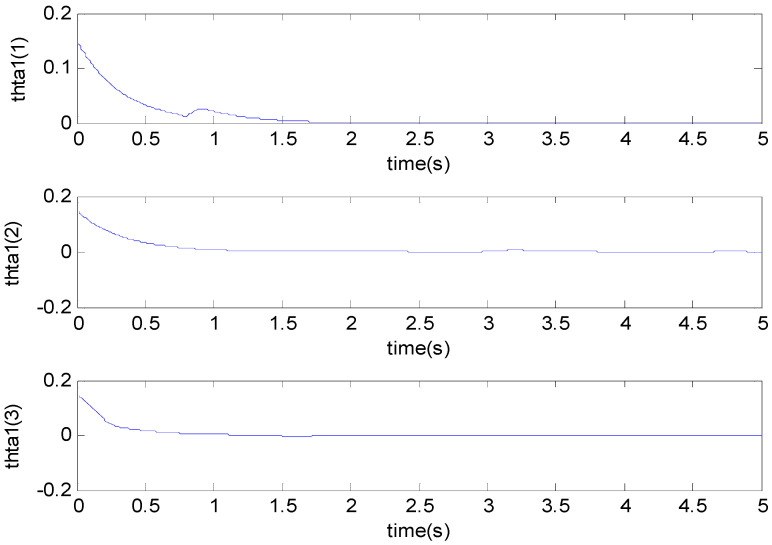
The first parameter using the ABFSMC approach.

**Figure 8:**
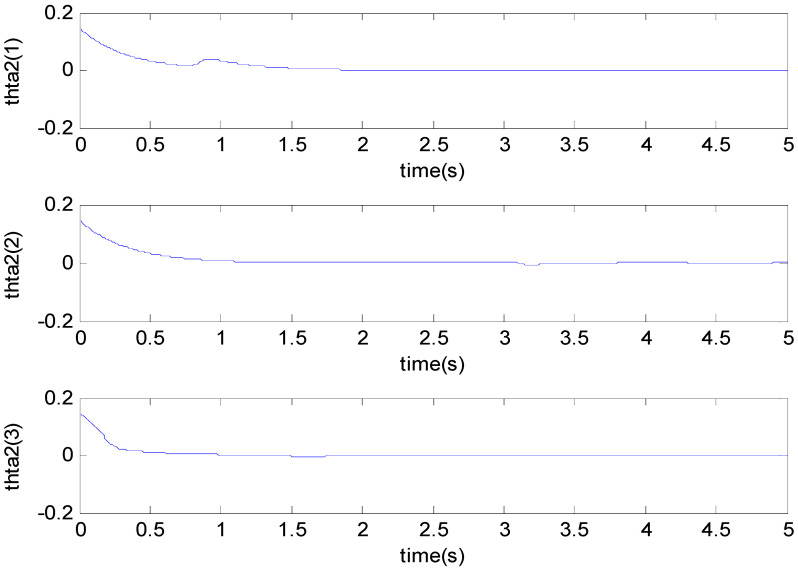
The second parameter using the ABFSMC approach.

Figure 3, Figure 4, Figure 5, Figure 6, Figure 7 and Figure 8 show the simulation results using the proposed ABFSMC approach. Figure 3 shows the output of micro gyroscope in the x−y axis to track the trajectory. Figure 4 shows the tracking error. It was demonstrated that the trajectory of the control system could track the reference trajectory in 0.2 s. The control input using the ABFSMC approach is drawn in Figure 5. It shows that the control input was stable between −1000 and 1000. Figure 6 shows the function of the sliding mode surface. The parameters of the fuzzy adaptive control are plotted in Figure 7 and Figure 8, showing that the fuzzy control method combined with the adaptive control method has the ability to learn and adjust the fuzzy parameters. It is observed that the fuzzy parameters can be adjusted to the optimal value quickly and keep stable, which shows that the self-regulation fuzzy system has better stability and self-tuning performance.

**Figure 9:**
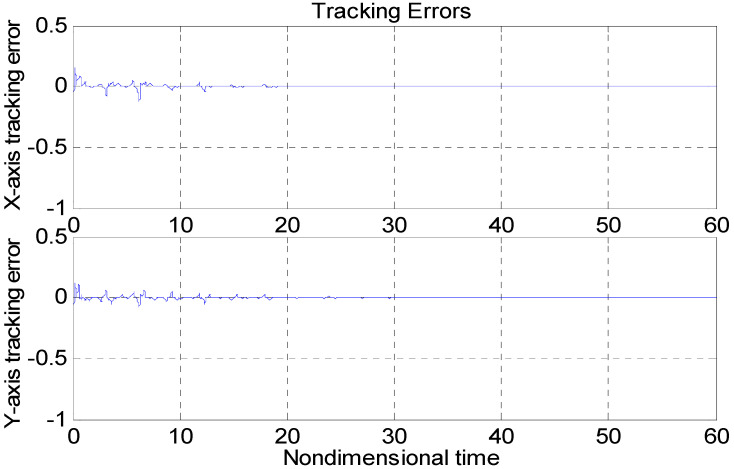
Tracking errors using the adaptive backstepping control approach.

**Figure 10:**
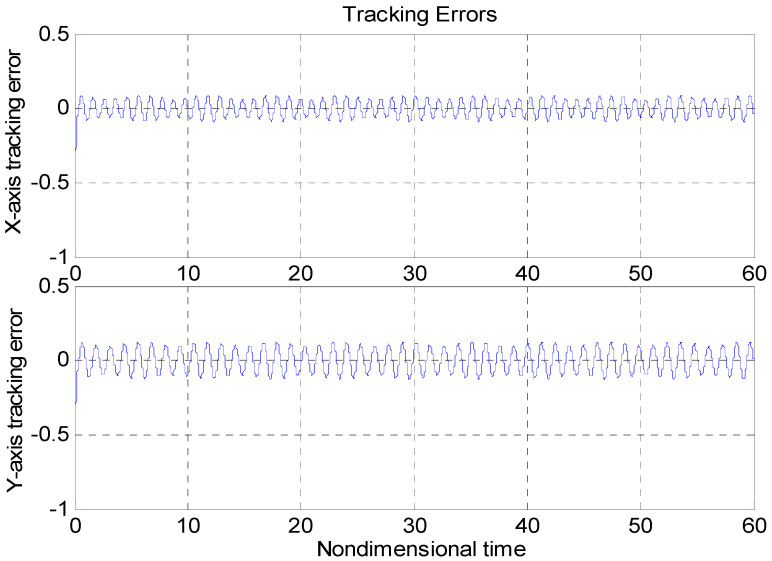
Tracking errors without using a controller.

An adaptive backstepping control (ABC) technique for a microscope was presented in [7]. In order to more clearly demonstrate the advantages of the proposed method, the performance of our proposed ABFSMC strategy was compared with the ABC method in [7] and the case without a controller. Figure 9 and Figure 10 show the tracking property using the adaptive controller in [7] and without using the controller, with the same nominal gyroscope parameters under the same model uncertainties. From Figure 10, due to the modeling error, the “dull” controller relied on the nominal parameters, which led to a stable system, but the tracking errors were obvious. The tracking errors with the adaptive backstepping controller displayed quite a large overshoot at the beginning, as did the control efforts. The settling time of the tracking errors was also worse than our proposed ABFSMC controller. The advantages of our proposed controller over the adaptive backstepping controller and the case without controller were obvious.

For the quantity discussion, the root mean square errors (RMSE) of the tracking error of the two axes of the micro gyroscope using these three difference controllers are compared in Table 2.

In summary, the introduction of the ABFSMC approach can adapt to the changes in the external disturbance and model parameters and maintain a satisfactory performance in tracking and approximation.

## 6. Conclusions

The ABFSMC strategy was investigated in a micro gyroscope through theoretical discussion and numerical simulation. The mathematical model of the micro gyroscope was transformed for the handiness of the backstepping control design. A backstepping approach was adopted to deal with the model uncertainties, disturbances, and unknown parameters of the micro gyroscope. The fuzzy parameters were updated online to approximate the nonlinear dynamics in the micro gyroscope. Simulation studies were investigated to demonstrate the advantages of the proposed ABFSMC strategy in tracking and approximation performance.

## Figures and Tables

**Table 1:** Parameters of micro gyroscope.

Parameters	Values
m	$1.8\times 10^{-7}\;\mathrm{kg}$
$k_{xx}$	63.955 N/m
$k_{yy}$	95.92 N/m
$k_{xy}$	12.779 N/m
$d_{xx}$	$1.8\times 10^{-6}\;N\;s/m$
$d_{yy}$	$1.8\times 10^{-6}\;N\;s/m$
$d_{xy}$	$3.6\times 10^{-7}\;N\;s/m$

**Table 2:** Root mean square errors (RMSE) of tracking error in *x*-axis and *y*-axis using three controllers.

Control Method	RMSE in *X* Axis	RMSE in *Y* Axis
ABFSMC approach	0.2356	0.3055
ABC approach	0.9845	0.8526
Without controller	7.9251	8.7976
